# Correlation with viruses enhances network complexity and stability of co-occurrence prokaryotes across the oceans

**DOI:** 10.1128/msystems.00539-25

**Published:** 2025-06-13

**Authors:** Bo Wang, Yantao Liang, Kaiyue Lian, Chuyu Zhang, Meiaoxue Han, Min Wang, Hongbing Shao, Andrew McMinn, Hualong Wang

**Affiliations:** 1 College of Safety and Environmental Engineering, Shandong University of Science and Technology 74789, Qingdao, Shandong, China; 2 UMT-OUC Joint Center for Marine Studies, Qingdao, China; 3 College of Marine Life Sciences, MOE Key Laboratory of Evolution and Marine Biodiversity, Frontiers Science Center for Deep Ocean Multispheres and Earth System, and Key Lab of Polar Oceanography and Global Ocean Change, Ocean University of China 12591, Qingdao, China; 4 Institute for Marine and Antarctic Studies, University of Tasmania 3925, Hobart, Tasmania, Australia; Colorado State University, Fort Collins, Colorado, USA

**Keywords:** marine viruses, prokaryotic microbiomes, network stability, community complexity, environmental gradients

## Abstract

Viruses play critical roles in shaping microbial distribution, organization, and ecological functions. However, the mechanisms behind the association and structuring of microbiomes with viruses still remain largely unknown. To understand how viruses affect the assembly of marine microbiomes, the network complexity and stability of prokaryotic microbiomes with/without viruses were analyzed and compared using the *Tara* Ocean Project data set, including samples collected from the Indian Ocean (IO), South Pacific Ocean (SPO), North Pacific Ocean (NPO), Mediterranean Sea, and South Atlantic Ocean. The network complexity of prokaryotic communities was substantially different across oceans, being high in the IO and low in the SPO and NPO. Network complexity and stability of marine prokaryotic microbial communities were enhanced by the influence of viruses: microbiomes associated with viruses were more complex, had a high modularity, and were robust with low fragmentation and vulnerability and higher compositional stability than prokaryotic microbiomes without an association with viruses. Results presented here shed light on how the associations with viruses influenced microbial assembly and their co-occurrence pattern and stability across the oceans. Overall, given such changed environmental characteristics across the oceans, stable and resilient microbiome networks imply that biotic associations with viruses play a critical role in maintaining the integrity and resilience of marine microbiomes and their ecological functions.

**IMPORTANCE:**

This study represents the first detailed research on the effects of viruses on the organization of marine prokaryotic microbiomes at the global scale. Biotic factors and environmental heterogeneity directly and indirectly affect microbiome and viral co-occurrence with varied strength. Network complexity and stability of marine microbial communities were enhanced by the influence of viruses. Thus, given such dynamic environmental gradients in the ocean, stable and persistent microbiome networks suggest that biotic associations with viruses play a critical role in maintaining the integrity and resilience of marine microbiomes and influencing the function of marine ecosystems.

## INTRODUCTION

Viruses are the most diverse and abundant biological entities across the oceans (1–3). There are approximately 10^10^ viral particles per liter of seawater, and most of them are phages (viruses that could infect bacteria) (4). Marine phages kill ca. 20% of the bacterial biomass each day (3) and thus shape microbial abundance, diversity and distribution and impact the biogeochemical cycling across global oceans (5). Viral lysis lessens the transfer of prokaryotic cells to protists, and the lysate released from the broken cells stimulates the growth of existing microbial communities, thus influencing the “microbial loop” (6). In addition, viruses can affect their hosts by horizontal gene transfer, selection for resistance, and manipulation of microbial metabolism (3, 7). Virus-associated microbial ecosystems represent excellent examples of complex marine ecological interactions, as they often contain thousands of different microbial types (6, 8–10). However, despite viruses having critical roles in controlling microbial distribution, organization, and functions, the mechanisms behind the association and structuring of these microbiomes remain largely unknown (11).

Defining the organization of microbiome and viruses and identifying the pattern behind their association are critical to clarify patterns of diversity, community relation and stability, and ecosystem functioning (11). Recent ecological and metagenomics studies have uncovered a wide range of viral diversity in marine environments, and viruses have infective specificities that range from broad to narrow (12). Viral-associated microbiomes are controlled by diverse mechanisms ranging from “ecological” drivers across the oceans, such as coevolution (7) and co-speciation (11). The adaptative changes of infectivity ranges of phages and hosts resilience are often explained as the coevolutionary “arms race,” in which hosts prone to broaden their range of resistance while phages tend to broaden their host range, such as *Synechococcus* sp. WH7803 and virus RIM8 (13) and the phage T4/T7 and *Escherichia coli* B (14, 15). Previous studies on the effects of viruses on microbiomes were potentially taxonomically or geographically restricted, focusing on specific abiotic factors (e.g., light limitation or nutrient availability) or targeting specific hosts and microbial groups. For example, lytic interactions between marine *Vibrio* bacteria and their phages are sparse, but recombination is common (16). Viruses are widely known to rapidly mutate and evolve, and under the proper conditions, viruses may switch from a specific to a broad host range, and even back (17). While broad-host-range marine phages have been identified in several cultivation-based studies, they are rare in bioinformatics studies (18–20). For more intimate associations such as antagonistic activities between viruses and bacteria, a strong community differentiation would be expected to be present across different marine environments.

To understand how viruses affect the association of marine prokaryotic microbiomes, multiple indicators can apply to characterize the community complexity and stability of microbial co-occurrence networks and their key members, such as robustness (21), vulnerability (22), fragmentation (23), node persistence (24), and compositional stability (25). A better clarification of the complexity and stability of virus-microbiome interaction networks is thus critical to identify basic patterns in their association and ecological functions. In addition, understanding the association of viruses and microbiomes can provide critical insight into how viruses influence microbiome organization. For instance, if the community network of a microbiome with viruses tends to be more/less stable relative to a microbiome without viruses, this would determine whether virus assembly processes affect the relation and network stability of prokaryotic microbiomes.

Here, we use the *Tara* Ocean Project data set (26), comprising almost 200 virus and prokaryotic microbial community samples collected from different oceans, including the Indian Ocean (IO), South Pacific Ocean (SPO), North Pacific Ocean (NPO), Mediterranean Sea (MS), and South Atlantic Ocean (SAO). Ten community ecological networks (prokaryotic microbiomes with/without virus) were then constructed across the five oceanic provinces through a random matrix theory-supported approach (27). Multivariate regression tree (MRT) and piecewise-structural equation model (SEM) statistical analyses were applied to illustrate how marine environmental gradients contribute to influencing the distribution of prokaryotic microbiomes and viruses, respectively. In addition, niche breadth and the neutral community model were applied to show the assembly process of marine microbiomes and viruses. In addition, the stability of these complex ecological networks, together with community and environmental data, was analyzed to determine whether (i) the global network is organized into modules in which viruses with highly overlapping bacterial communities group together, suggesting potential commonalities of their organization, (ii) the environmental gradients directly/indirectly shape the assembly of marine prokaryotic microbiomes and viruses, respectively, and (iii) the community network complexity and stability were significantly increased by including the association with viruses.

## RESULTS

### Effects of environmental gradients on the composition of viruses and prokaryotes

The environmental parameters were compared and varied across five sea areas. For example, the SAO exhibited significantly lower temperatures than the IO (*P* < 0.01), while the MS showed significantly higher salinity compared to other regions (*P* < 0.01) (Fig. S1). The nutrient availability and the abundance of bacteria also varied across the oceans. The distribution of prokaryotic and viral communities was clustered into different oceanic provinces that gradually changed with temperature across the oceans (Fig. S2). Similar patterns were also found when only those samples including both prokaryotes and viruses were used for the statistical analysis. The MRT analysis showed that depth, dissolved oxygen, temperature, and geographic gradients were the main drivers shaping the spatial variations in prokaryotes and viruses (Fig. S3). In summary, the accumulative variance in prokaryotic microbial communities was better explained by depth (18.9% vs 6.5%) and dissolved oxygen (10.8% vs 9.7%), while temperature (4.68% vs 8.56%) better explained the viral distributions (Fig. S3). Given numerical ranges of environmental parameters generated from MRT have provided critical information for classifying viral and microbial community distribution into different groups to better clarify the classification of environmental variations on prokaryotic and viral distribution. The redundancy analysis (RDA) and Mantel test analysis results further demonstrate that the environmental gradients made a great contribution to the spatial distribution of prokaryotic and viral communities and explained why the viral variation (25.0%) was less than that of the prokaryotic microbial communities (41.3%) (Fig. S4 and S5). Thus, biotic factors (including the total abundance of picoeukaryotes, autotroph prokaryotes, and heterotroph prokaryotes, respectively) and nutrient availability contributed greatly to the prokaryotes distribution, while temperature and latitude shaped the distribution of the viral community. In general, these results demonstrate that environmental heterogeneity shaped the viral and microbial community distribution with varied strength across the oceans.

**Fig 1:**
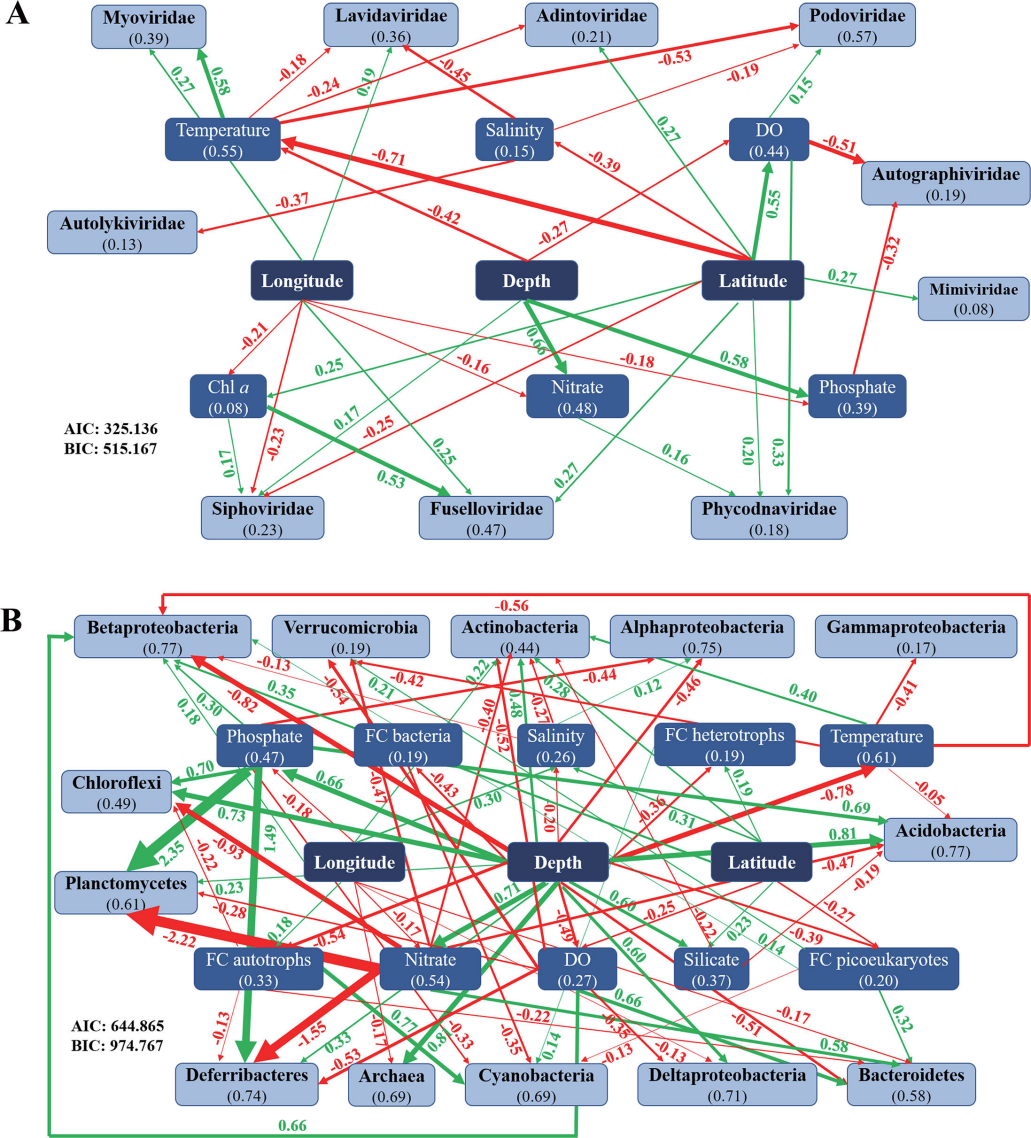
Piecewise-structural equation model to demonstrate the direct/indirect relations between environmental gradients and distribution of viruses (**A**) and their surrounding microbiomes (**B**), respectively. Red lines, green lines, and the associated numbers indicate significant negative or positive correlations and the standard estimates. The thickness of arrows and the number associated with each line represent the strength of correlations. Numbers in parentheses show the explanation degree for each factor.

Direct and indirect effects of environmental variations relative to major viral and prokaryotic microbial groups in the oceans and the level of multiple effects were analyzed and compared by piecewise SEM (Fig. 1). Latitude and depth made the greatest contributions to the composition and associations of major viral groups and also strongly influenced other environmental factors including temperature,dissolved oxygen (DO), salinity, Chl a, and nutrient availability (Fig. 1A). For instance, increasing latitude was associated with increased abundance of Phycodnaviridae, Mimiviridae, Fuselloviridae, and Adintoviridae (positive effect), and decreased abundance of Siphoviridae (negative effect) (Fig. 1A). Through relations with temperature and Chl a, the latitude also affected other viral groups such as Myoviridae and Lavidaviridae (Fig. 1A). Along with temperature, DO, nitrate, and phosphate, depth gradients impacted several virus groups including Myoviridae, Podoviridae, Autographiviridae, and Phycodnaviridae (Fig. 1A). Similarly, the microbial communities in the ocean were shaped by the direct and indirect influences of environmental heterogeneity with much more complex relations compared to those of viruses (Fig. 1B). For example, enhanced depth and decreased longitude were associated with increased Deltaproteobacteria and Archaea, while Actinobacteria exhibited an inverse relationship. In general, the results clearly show how spatial gradients in environmental factors affect marine viruses and microorganism associations.

### Relative importance of stochastic processes to community assembly

**Fig 2:**
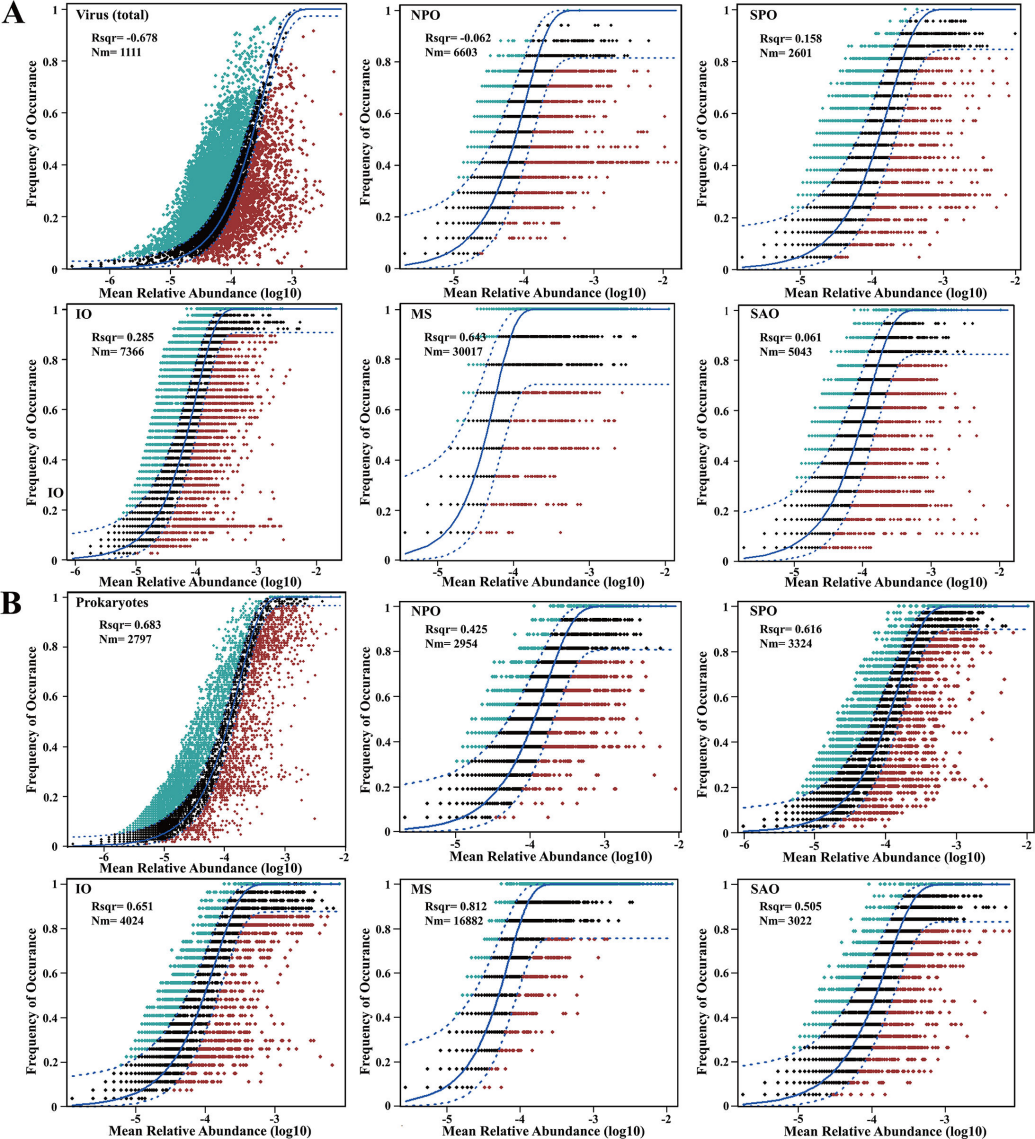
Fit of the neutral community model for viruses (**A**) and surrounding microbiomes (**B**) across the oceans. The solid blue lines represented the best fit to the model, and dashed blue lines indicated the 95% confidence interval around the model prediction.

Thebeta-nearest taxon index (βNTI) analysis results showed that deterministic processes play key roles in shaping the assembly of prokaryotic microbiomes across the oceans, while the relationships between the occurrence of(viral) operational taxonomic units ([v]OTUs) and their relative abundance have been well described by the neutral community model (Fig. 2; Fig. S6). The stochastic processes influenced greatly and almost equally the assembly of viral and prokaryotic microbial communities (Fig. 2). In addition, the relative effect of stochastic processes to the organization of the viral community was greater in the MS (64.3%), while the stochastic processes explained less of the variation in the viral community in the IO (28.5%), SPO (15.8%), NPO (6.2%), and SAO (6.1%) (Fig. 2A). A similar pattern was also observed in the prokaryotic microbial communities. But with greater strength, the relative effect of stochastic processes decreased gradually with the rank of the MS, IO, SPO, SAO, and NPO (Fig. 2B).

**Fig 3:**
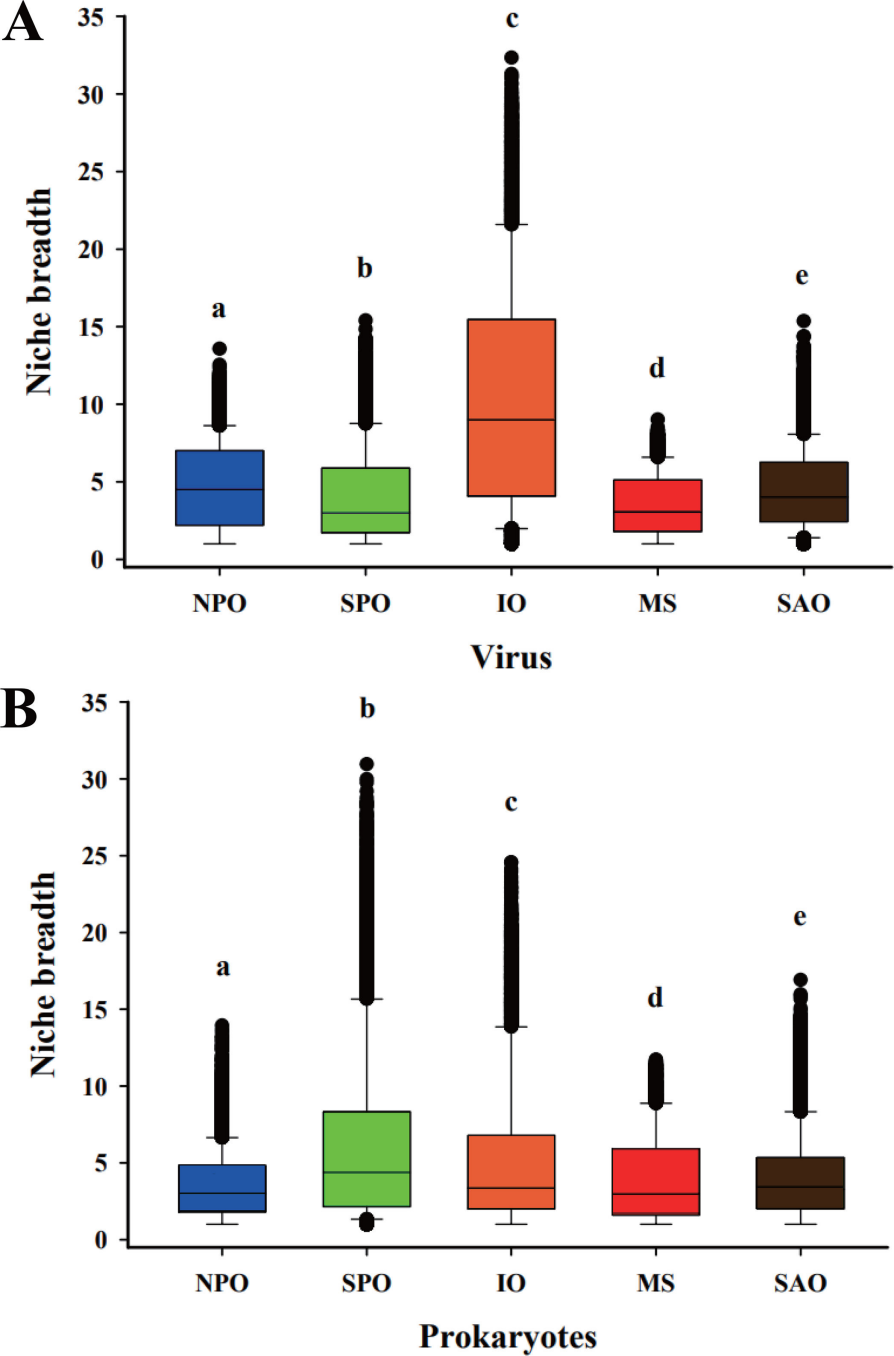
Environmental niche breadth of viruses (**A**) and microbiomes (**B**) across the oceans. Different letters above the bars indicate significant differences among major oceanic provinces (*P* < 0.01), while shared letters indicate no significant difference.

The environmental niche breadth of the viral community was strongly different across the oceans (*P* < 0.01) and decreased sequentially from the IO to NPO, SAO, SPO, and MS (Fig. 3). Although the niche breadth of the prokaryotic microbial community was also significantly different across the oceans (*P* < 0.01), the niche breadth was significantly higher in the SPO and IO than in the SAO, MS, and NPO (*P* < 0.01). These results show that spatial gradients influence the assembly process and environmental niche breadth of viruses and prokaryotic microbiomes with varied strength across the oceans.

### Co-occurrence network complexity of viruses and microbiomes

**Fig 4:**
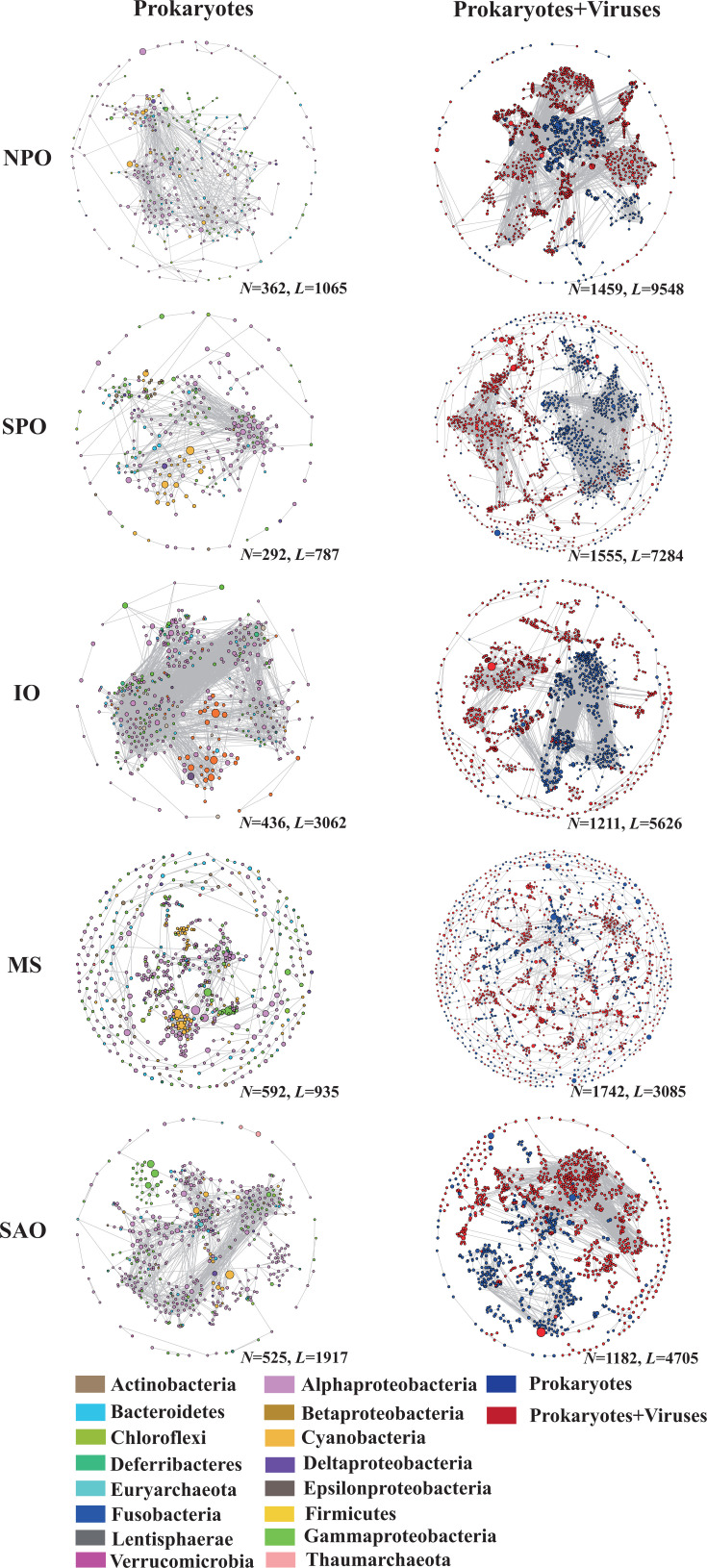
Co-occurrence networks of microbiomes with/without viruses across the oceans. Color-coded nodes represent major microbial groups of prokaryotes and viruses in different networks. The degree of each taxon is shown by node sizes. *N* represents the total number of nodes, and *L* represents the total number of links in each specific network.

Microbial community co-occurrence networks were constructed based on all data sets from the NPO, SPO, IO, MS, and SAO, respectively (Fig. 4). Network size (total amount of nodes) of the microbial communities was greatest in the MS (592) and lowest in the SPO (292), while the network connectivity (total amount of links), average connectivity (average degree, average links per node), average clustering coefficient (the degree to which nodes are clustered), graph density, and the number of positive links were high in the IO and low in the SPO/MS (Table S1). These results show that the network complexity of microbial communities was substantially different across the oceans due to the environmental heterogeneity; it was highest in the IO (more connections) and lowest in the MS.

To further clarify how viruses affected microbial community network complexity, network topology changes of microbiomes with an association with viruses were calculated (Table S1). Including the association with viruses enhanced the network complexity of microbial communities across all oceans (Table S1). In addition, the number of nodes, links, average degree, graph density, modularity, and positive links were high in the network of samples from the SPO and NPO, although the modularity and nodes were also high in the network of MS. Thus, after including the biotic relations with viruses, the network complexity of microbial communities was increased and turned into a distinct pattern across all oceans. The number of positive and negative pairwise correlations can reflect the degree of cooperative and antagonistic behaviors of microbiomes with/without viruses in a specific oceanic province, and these results suggest that microbial connectedness and hence microbial interactions were enhanced in the virus-abundant environments.

Changes in network characteristics of microbiomes with/without a relationship with viruses were also affected by community organizational principles, such as the modularity (Fig. S7). The network modularity and the number of connected components and clusters of microbial communities were greater in the MS and low in the IO (Table S1). Interestingly, after including the relationship with viruses, the modularity and the number of connected components and clusters were high in the MS but low in the SAO and NPO, respectively, although the levels of these matrixes were all enhanced. These results indicate that an association with viruses significantly enhanced the network modularity of microbiomes but with varied strength across the oceans.

The altered community network complexity could result in changes in the role of specific members within each network (Tables S2 and S3). The amplicon sequence variants (ASVs) that were affiliated to the SAR11 clade and Cyanobacteria were the main top 10 nodes with a high degree/betweenness (Table S2). In addition, on the basis of those nodes with top 10 Pi (the connectivity among network modules) and Zi (the connectivity within network modules), diverse microbial taxa were identified across these microbial community networks including SAR11, Oceanospirillales, Rhodospirillales, and Rhodobacterales (Table S3). Those nodes with high either degree/betweenness or Zi/Pi can be regarded as key nodes that play critical roles in shaping community network topological structure and hold the network together across the oceans.

### Effects of virus on the network stability of co-occurrence microbiomes

**Fig 5:**
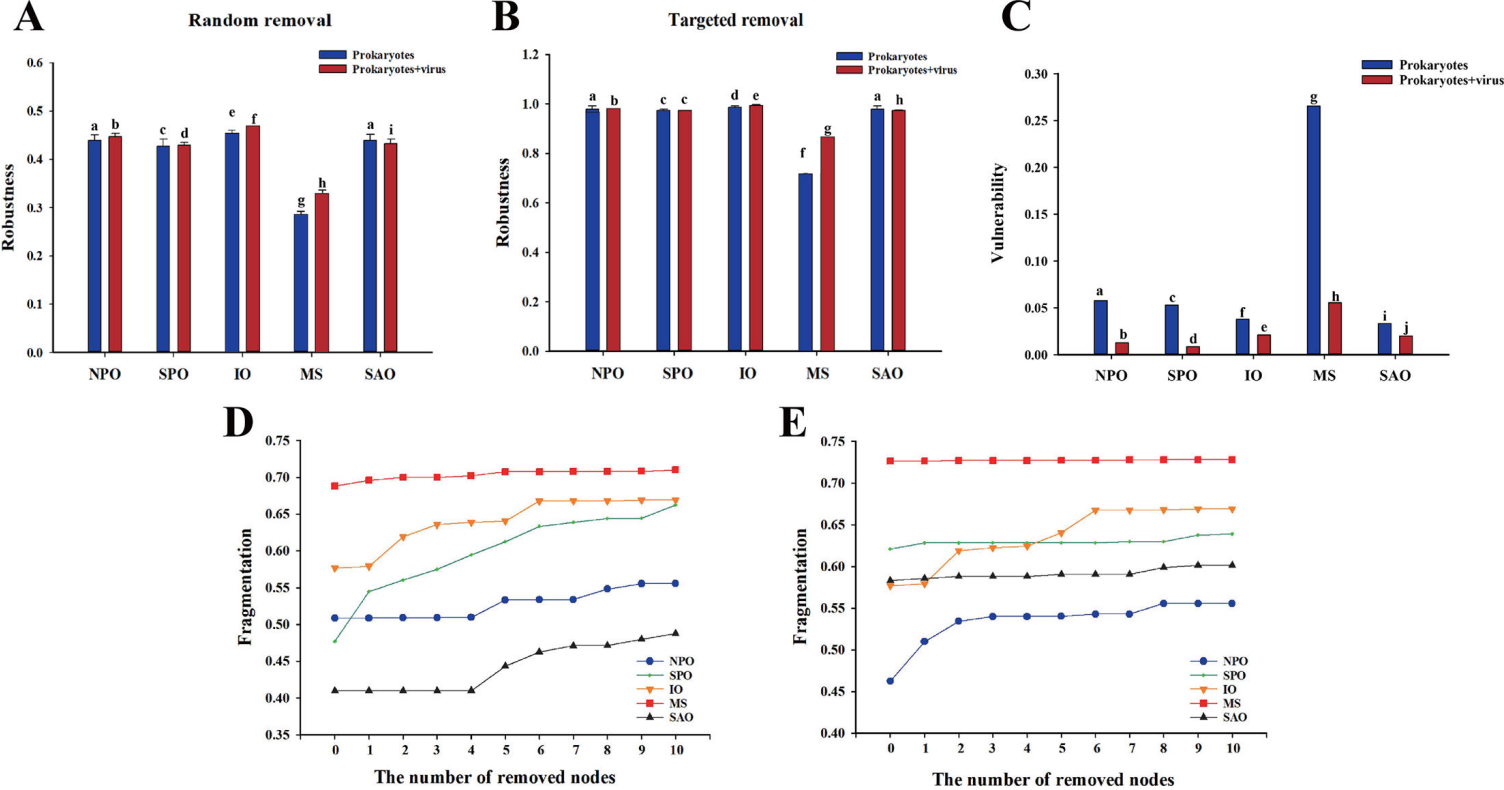
Network robustness, vulnerability, and fragmentation of microbiomes with/without viruses across the oceans. (A) Robustness measured as the proportion of taxa remained with 50% of the taxa randomly removed from each of the community networks. (B) Robustness measured as the proportion of taxa remained with five module hubs removed from each of the community networks. (C) Network vulnerability measured by maximum node vulnerability in each network. Different letters above the bars indicate significant differences among major oceanic provinces (*P* < 0.01), while shared letters indicate no significant difference. (D) The network fragmentations of co-occurrence prokaryotes with consecutive removal of 10 nodes with the highest betweenness centrality. (E) The network fragmentations of co-occurrence prokaryotes with viruses.

To further explore the influences of virus on the microbial community stability, the network robustness, vulnerability, fragmentation, nodes persistence, and the compositional stability were calculated and compared (Fig. 5). First, species extinction and then calculated robustness of the microbial community networks were simulated. On the understanding that either targeted removal of network hubs or random species loss ( including viruses) could alter stability, we found that these processes instead enhanced microbial community network robustness in each ocean (*P* < 0.01) (Fig. 5A and B), except in the SPO. Furthermore, the network robustness of microbiomes with/without the association with viruses was significantly different across the ocean, respectively. The network robustness of microbiomes with/without viruses was all significantly lower in the MS than in other oceans.

Network vulnerability of microbiomes with/without the association with viruses was varied across the oceans (*P* < 0.01) (Fig. 5C). Relationships with viruses significantly weakened the vulnerability of microbiomes (*P* < 0.01). Interestingly, the network vulnerability of microbiomes with/without viruses was higher in the MS than in other oceans, and it was in contrast to the pattern of robustness. The network fragmentation of microbiomes with/without the relation with viruses was distinct across the oceans, and it was high in the MS and low in the NPO and SPO (Fig. 5D and E). In addition, the fragmentation of microbial communities was decreased after including the relationship with viruses in the SPO and SAO in terms of the increment after removing those key nodes, while it remained unchanged in the ocean MS and IO.

**Fig 6:**
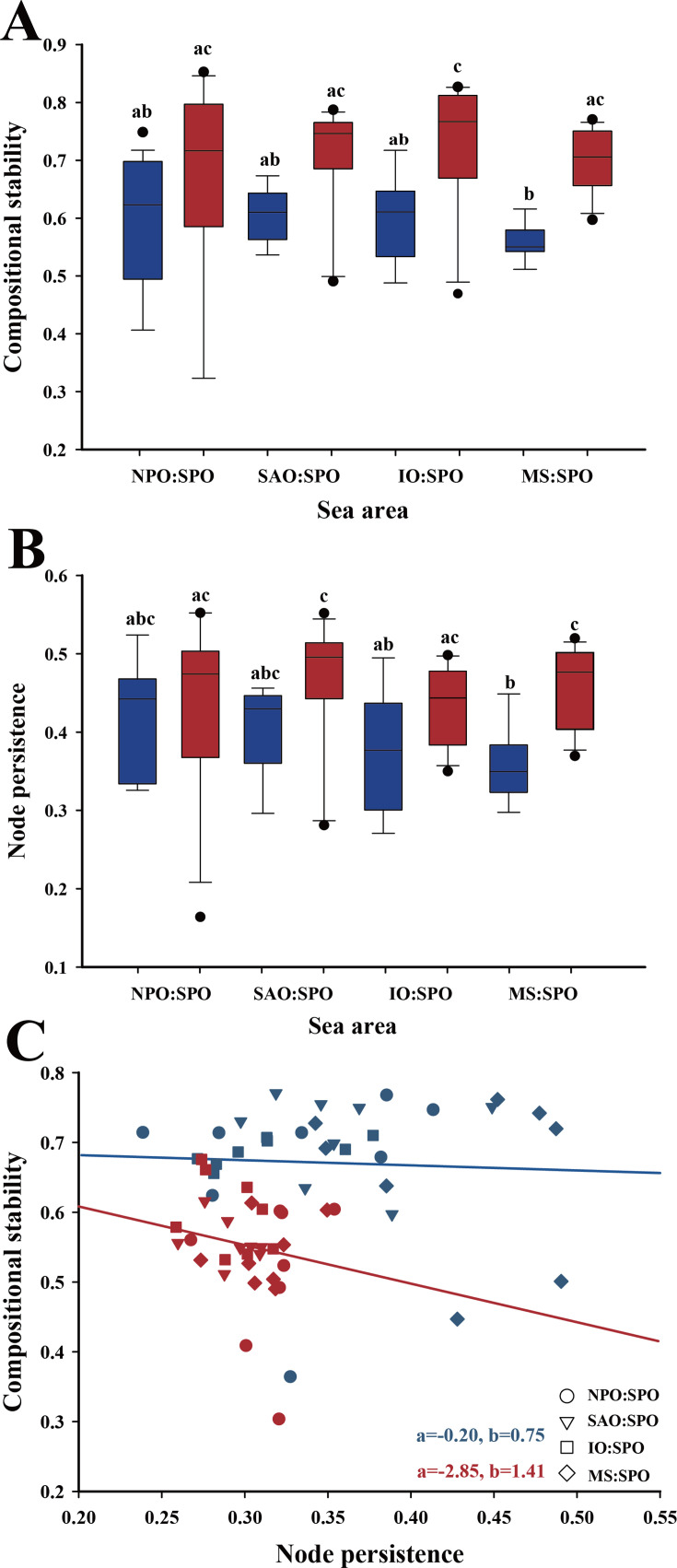
Community network stability of microbiomes and viruses across the oceans, respectively. (**A**) Compositional stability. (**B**) Node persistence. (**C**) The relation between compositional stability and node persistence. Blue color represents microbiomes, and red color represents microbiomes with viruses. Different letters above the bars indicate significant differences among major oceanic provinces (*P* < 0.01), while shared letters indicate no significant difference.

Multiple network stability parameters calculated from empirical data showed that the network stability of microbiomes increased after including the relationship with viruses. First, multi-order microbial compositional stability increased after including the relationship with viruses (Fig. 6A), while the combined viral and microbial community networks had more nodes, links, and modules. Similar patterns were also found in the variation of node persistence that was higher after the inclusion of the relationship with viruses (Fig. 6B). In addition, node persistence was strongly related to the compositional stability across all samples (Fig. 6C). These results indicate that microbial community composition became stable when including the biotic influence of viruses, and the community network complexity was enhanced.

## DISCUSSION

### Association with virus-enriched microbiome network complexity

Influence of viruses significantly stimulates the correlation of microbiomes, particularly the number of components, clusters, and positive/negative links in the community networks, which leads to enriched community complexity, and hence, the linked microbiome ecosystem functions could be more complex in a virus infection efficiency world (28). Negative frequency-dependent selection drives diversification of phages (to track their co-evolving hosts) and hosts (to escape phages), thereby creating a diverse microbiome community through kill-the-winner microbial ecological dynamics. For example, viruses structure marine microbial diversity and dynamics by causing ca. 10^28^ new infections of their microbial hosts each day in the oceans (3, 29). In addition, significantly more frequent virus-host co-occurrence relations were observed in seawater than in sediment (30). The lytic cycle is the preferred mode of existence for plankton viruses inhabiting a nutrient-abundant marine environment, in sharp contrast to the lysogenic lifestyle of their deep-sea counterparts (31).

The network complexity of microbial communities was substantially different across oceans, being high in the IO and low in the SPO and NPO. Meanwhile, the interactive IO could influence the El Niño-southern oscillation cycle and also influences the heat flux anomalies in the surface IO and surface wind stress anomalies in the tropical Pacific and Indian Oceans (32). Thus, these results suggest that small- and large-scale marine processes play critical roles in structuring microbial and viral community composition and associations. For example, the Agulhas current and subsequent ring formation (33) link viral communities between the Atlantic and Indian Oceans, as also found in planktonic communities from the *Tara* Oceans expedition (26). These abundant communities then form the “seed bank” in neighboring environments and are passively transported through ocean currents, and the evolutionary rate varied across size. For example, the smaller plankton microorganisms (<20 µm) had a shorter metagenomic turnover time than greater plankton in the North Atlantic Ocean (including the Mediterranean Sea).

Viral associations increased microbiome network complexity, indicating that broad-host-range viruses are probably distributed widely in the oceans (18). For example, multiphylum-infecting phages (such as ɸFenriz and ɸVader) are capable of infecting five strains belonging to different phyla including Proteobacteria, Bacteroidetes, and Actinobacteria (34). Metagenome-based research and the clustered regularly interspaced short palindromic repeats matches have also found viral clusters linked to multiple microbial hosts from different phyla (35). Pleiotropic trade-offs between virulence and microbial host range may lead to broad-host-range phages with low growth rates being defeated by narrow-host-range phages with greater virulence. Some of the cross-class or even cross-phylum infectivity was further demonstrated by host prediction based on the virus-host homology analysis results of CRISPR spacer or other critical nucleotide sequence (30). In addition, significantly intertwined coinfections among temperate viral clusters were calculated by analyzing their co-occurrence characteristics in the same host, especially in seawater, indicating their strong compatibility and potential mutual effects.

Larger-scale community networks often show modularity structures (36), such as viruses and their hosts across the oceans. Modular co-occurrence networks may be more likely in the heterogeneous marine environments, where viruses and microbes interact in subsets. Based on our piecewise SEM statistical results and earlier studies (37), it can be seen that large-scale dynamic patterns of marine viruses are affected by microbial host density and diversity and causing environmental factors. For example, viral density in the upper 200 m of the marine water column is great in (sub)tropical areas but lower in Antarctic areas (37, 38). Within marine microbiome networks, associations between taxa can be driven by two critical mechanisms: environmental filtering and species interactions (39, 40). In the case of microbial species interactions, positive correlations can be caused by facilitation/mutualism among taxa, and negative correlations can result from competition (41, 42). In the case of environmental filtering, positive relations among taxa represent ecological or functional similarity, while microbial and/or viral species with divergent niche requirements are negatively correlated (43).

### Microbial community robustness and vulnerability influenced by the relation with viruses

Enhanced network robustness and stability of microbial communities were observed after including the association with viruses across the oceans. Environmental adaptation and coevolution between hosts and their phages contribute to the varied community robustness and vulnerability of microbiomes across the heterogeneous oceans. For example, the relation with viruses enhanced microbial robustness in the ocean NPO, IO, and MS, while the vulnerability was decreased. The outcome of microbial and viral interactions depends on the balance between the benefits and costs provided to the partners. Mutualistic and antagonistic interactions, which lead to a loss of partners, can enhance marine microbial community robustness, but this enhancement decreases when the structure is progressively disrupted (21). In virus-microbiome networks, a modular structure can show common community organization, while the removal of network “gatekeepers” disproportionately contributes to community network fragmentation, which has potential influences for the functions microbiomes fulfill in the marine ecosystems (23). For example, virus-host coevolution in the marine environments influenced their genomic features, such as the GC content of seawater temperate viruses first enhanced and then decreased along water depth, while the carbon atoms showed an opposite trend (30). Virus-encoded reverse dissimilatory sulfite reductase was found in hydrothermal vent plumes from the Southwest and East Pacific Ocean (such as Lau and Guaymas basins), suggesting that viruses play key roles in the oceanic element cycling and the microbial loop (44).

Enhanced stability with complex co-occurrence patterns between microbiomes and surrounding viruses supports that the resistance of marine microbiomes to viral infection varied across major groups. Some microbial hosts possess complete resistance to the viral infection, although others are entirely susceptible or show susceptibility to specific strains (45, 46). For example, different groups of marine microbial communities showed divergent lysogeny ratio (LyR), ranging from 8% (Thermococci) to 91% (Desulfovibrionia), and bacteria showed significantly higher LyRs (41%) than archaea (29%) at the class level (30). Many *Prochlorococcus* species and their phages have been identified with many relationships in microbial community networks across the oceans. For example, the resistance-evolving process of *Prochlorococcus* could increase their growth rate and decrease resistance range over generations (47). The resistance property of microbial hosts can be contributed by CRISPR-Cas systems (48), which were widely found in up to 90% of archaea and nearly 45% of microbiomes and has yet to be properly investigated in the oceans (49).

Diverse communities and complex associations between microbiomes and surrounding phages are shaped by their evolving arms race (50). Host resistance to viruses can exact a pleiotropic fitness consumption, such as decreased growth rate or enhanced vulnerability to infection by other viruses (51). In phage–host coevolutionary researches, increased host range of a phage is often accompanied by decreased virulence level of that phage on each specific host, such as the nontailed *Autolykiviridae* (*Vibrio* phages) (20). Meanwhile, hosts that evolve resistance to one specific phage can become susceptible to another one in complex phage–host systems (52, 53). Given that costs of generalism are found in many ecosystems, arms races may generally give way to fluctuating selection in the antagonistically co-evolving populations (51). As a result, environments with high viral stress will have a greater effect on the shared marine ecological niche (e.g., replacement of compatible microbes with incompatible microbes, destruction of the marine ecological community, etc.) of microbial taxa than on marine environments with low viral stress.

### Association with viruses enhances the network stability of marine microbiomes

In this study, it was found that association with viruses stabilized marine microbial community networks: microbes associated with viruses were more complex and modular and mainly dominated by high robustness and strong compositional stability and low vulnerability compared to only microbial community networks. The broader biogeography of these relations shows the interconnected evolutionary process of microbiomes and viruses and their pervasive role in shaping fundamental processes such as nutrient regeneration, primary production, and biogeochemical cycling across the oceans (54). For example, dimethylsulfoniopropionate serves as both a chemical signalization molecule involved in microbial associations in the ocean and as a sulfur and carbon source preferentially consumed by abundant microbial taxa, such as SAR86, SAR11, and Rhodobacterales (55). Viruses could shape the taxonomic and functional composition of microbiomes, for instance, through “kill-the-winner” dynamics that maintain abundance and diversity (11).

Enhanced resistance of marine microbiomes occurs in virus-enriched environments, although co-occurrence networks imperfectly reflect coevolutionary dynamics (56). Consistent results have also been observed in previous community studies (57). Virally encoded auxiliary metabolic genes (AMGs) in the oceans appear to be related to nitrogen, sulfur, and other critical elements cycling (19), and thus steer microbial metabolic pathways, such as nutrient acquisition and photosynthesis, and then promote viral replication. For example, approximately 88% of cyanophage genomes were found to possess photosynthesis genes (58), and these genes were widely observed in sipho-, myo-, and podoviruses in the oceans. The *Synechococcus* phage Syn9, which infects multiple environmental isolates, maintained a uniform transcriptional program despite eliciting different responses across three hosts (59). The virulent phages could drive density- and frequency-dependent dynamics of microbiomes, where they suppress the most common microorganism, enabling minority populations to rise in frequency (11). Therefore, preserving microbial “interactions” with viruses could be important in mitigating the detrimental effects of community diversity loss on marine ecosystem functions (60).

Global dispersal capacity for microorganisms and corresponding environmental selection strongly shaped the association and co-occurrence stability of microbiomes and viruses (61). Plankton microbiomes show a greater similarity of co-occurrence communities within than across ocean regions (62, 63). Enhanced microbial richness with temperature was observed from 4°C to ca. 12°C, and followed by a negative relation (up to 30°C) (63). Global warming could result in the tropicalization of the composition and diversity of planktonic groups and may have multiple influences on marine microbial and viral associations and functioning and is expected to be specifically significant in critical areas for fisheries, carbon sequestration, and marine conservation.

Higher connectivity among microbiomes would be related to enhanced ecological robustness and stability, with highly connected microbial communities being more resilient and persistent. Results presented here illustrate how the complexity of viral interactions with their hosts may allow diverse microbiomes to coexist within a shared marine environment and highlight the ecoevolutionary roles of viruses in the marine microbial ecosystems (57). Plankton microbiomes will be influenced differently by environmental heterogeneity in specific oceans and future perturbations, such as global warming and nutrient distributions (64). Each resulting marine microbial network represents a snapshot of the microbiome that can be used to characterize potential interaction patterns and to predict stability and be comparable to other networks similarly constructed (65, 66). Although co-occurrence networks between prokaryotes and viruses cannot fully capture their ecological interactions, our network stability analyses reveal patterns of microbial community stability across the oceans and establish testable hypotheses for future experimental validation. In addition, although the enormous genetic diversity of viruses across the oceans has been demonstrated in multiple fundamental data sets (30, 35, 67), typical viruses and their hosts warrant isolation, characterization, and molecular mechanism research to reveal details of their association and ecological functions.

### Conclusion

This study implies that variations in viral abundance dictate stable and resilient marine prokaryotic microbiome associations across the oceans. An association with viruses enhanced prokaryotic microbiome network complexity and stability. Understanding how the complexity and stability of co-occurrence prokaryotic microbiomes are impacted by biotic influences of viruses has critical implications for modeling species’ distributions, maintaining and protecting ecosystem processes and functions. The piecewise SEM results demonstrated that biotic factors and environmental heterogeneity directly and indirectly affect prokaryotic microbiome and viral co-occurrence with varied strength, and these variations manipulate the stability and tolerance/susceptibility of marine virus-microbiome interaction. As anthropogenic disturbances increasingly stress marine ecosystems, the community of microbes and its tightly related viruses may become further destabilized.

## MATERIALS AND METHODS

### Sample characterization and sequence assembly

All microbial and viral sequence data, along with environmental parameters, were retrieved from the *Tara* Oceans OM-RGC (Ocean Microbial Reference Gene Catalog) (https://ocean-microbiome.embl.de/companion.html) and associated publications (12, 26, 63, 68). Briefly, during the *Tara* Oceans expedition, seawater was collected from 5 to 990 m across major oceanic areas (Fig. S1), including the SAO, SPO, NPO, IO, and MS, for prokaryotes (108 samples) and viruses (75 samples) community analysis. The environmental parameters and the microbial and viral sampling and sequencing data were originated from the same samples, and these were performed by the *Tara* Oceans team. Details of sample collection, methodology, and environmental data have been described by *Tara* Oceans previously (69–72).

Metagenomic DNA from prokaryotes and viruses was extracted, sequenced, and assembled as previously described (12, 63, 68). Briefly, DNA (30 to 50 ng) was sonicated, end-repaired, and 3′-adenylated before Illumina adapters were added. Ligation products were purified, PCR-amplified, and size selected (~300 bp) on a 3% agarose gel, and then each library was sequenced on Illumina sequencing machines (Illumina, USA). High-quality (HQ) reads were generated and assembled using MOCAT (version 1.2). After mapping all HQ reads against representative genomes which were selected from publicly available ocean metagenomic data and reference genomes (63), resulting in a total of 137.5 M gene-coding nucleotide sequences and 40,154,822 genes. Overall, these processes resulted in a global OM-RGC, which comprises >40 M nonredundant representative genes from viruses, prokaryotes, and picoeukaryotes. The microbial and viral abundances and environmental data were retrieved from the website of *Tara* Oceans (http://ocean-microbiome.embl.de/companion.html), and the viral classification was updated to blast against the IMG/VR 3.0 database (73).

### Distribution of marine microbiomes and viruses and causing environmental factors

Nonmetric multidimensional scaling (NMDS) was performed by Bray-Curtis distance matrices in order to describe variations in microbial and viral communities. The NMDS analyses were conducted using “metaMDS” in the vegan package (R version 4.1.1). To determine how the microbiomes and viruses respond to environmental gradients, MRT analysis was applied to evaluate the hierarchical effects of the spatial gradients on the communities (74). It was generated by the package “mvpart” (R version 4.1.1.), and the divisions were calculated by cross-validation.

RDA was applied to explore the relations between environmental factors and the microbial and viral communities by the vegan package, respectively. This analysis was calculated from marine environmental variables data and the Hellinger-transformed abundance data (75). Forward selection with the “ordistep” function in the package “vegan” was used to obtain the “best” explanatory variables and to identify when to stop the selection. The significance of the RDA statistical analysis results was calculated by analysis of variance through 999 permutations. This statistical analysis helped to determine the most influential parameters and to what extent these environmental factors shaped microbial and viral composition.

The piecewise SEM (73) was applied to analyze the hypothetical pathways (directly and indirectly) that explain how spatial variations shaped the composition and associations of microbiomes and viruses, respectively. Compared to RDA, it allowed the separation of direct and indirect influences of each environmental factor relative to major microbial/viral groups and to estimate the degree of multiple effects. From the MRT analysis and the RDA results, longitude, latitude, and depth gradients were identified as the major driving factors. These three environmental factors covered the spatial variation across the oceans and therefore shaped microbial and viral associations through changing temperature, Chl a, and nutrient availability (e.g. N, P). Piecewise SEM was calculated in R (version 3.6.1) through the “piecewiseSEM” package.

### Neutral community model and niche breadth of microbiomes and viruses

To determine the potential contribution of stochastic processes on the assembly of microbiomes and viruses across the oceans, a neutral community model has been applied to calculate the relationships between the taxa abundance and their detection frequency (76). All computations were calculated in R package “Hmisc,” “minpack.lm,” and “stats4” (version 4.1.1) (77).

Niche breadth represents the sum of the diverse resources that a taxon uses (78). To identify the taxa sorting and dispersal patterns, the Levins’ niche breadth index based on Shannon-Wiener index (79) for microbiomes and viruses was estimated according to the formula as previously described (80). The analysis was conducted using the R package “spaa” (81).

### Co-occurrence network analysis

Co-occurrence networks of microbiomes with/without viruses were constructed by therandom matrix theory (RMT)-based network tool (22), and it is available on the website of the University of Oklahoma (http://ieg4.rccc.ou.edu/MENA/). To reduce potential biases in correlation coefficients caused by sparse ASV matrices, only ASVs detected in >65% of samples with similarity correlation coefficients exceeding 0.88 were included in network construction. Network topological analysis and visualization were performed in Gephi (version 0.9.2). The topological characteristics of the community networks were analyzed with indexes, including degree (number of connections), components (separated subgraphs), average clustering coefficient (the level that nodes are prone to cluster together), modularity, network diameter (longest distance), clusters, graph density, average path length, proportions of negative and positive correlations, and network fragmentation (*f*) (82–85). The nodes with a high degree or high betweenness centrality are critical for microbial network pattern and persistence because they actually hold the network together (86–89).

Network complexity (including network size, average clustering coefficient, connectivity, relative modularity) and network stability (including vulnerability, robustness, fragmentation, compositional stability) of microbiomes with/without viruses were calculated and characterized. The *f* was performed as the ratio of disconnected subgraphs (CL) to the number of nodes (N) in each community network as log(CL)/log(N) (23). The *f* ranges from 0 to 1; those closer to 1 represent less stable and more fragmented networks. Loss of “gatekeepers” (with high betweenness) contributes disproportionately to community network fragmentation, showing high fragility of microbial community networks upon removal of specific taxa (23, 90).

The robustness of a network is calculated as the ratio of the remaining microbial taxa in this co-occurrence network after targeted or random node removal (21, 91). The ratio of the remaining number of nodes was defined as the network robustness. The robustness was calculated when five module hubs or 50% of random nodes were removed. The vulnerability of each microbial taxon measures the relative contribution of the taxa to the global community efficiency. The vulnerability of a microbial community network is calculated by the maximal vulnerability of taxa in the network, and the global community efficiency was measured as the average of efficiencies across all pairs of nodes. The efficiency shows how fast information/influence spreads within the network.

Node persistence is calculated as the ratio of coexisting taxa (over the total amount of taxa) in an ecological oceanic province (92). The node persistence, as the ratio of nodes occurring in the community network across the oceans, was thus calculated. Compositional stability indicates the variation of the structure of microbiomes with/without viruses across the oceans (93, 94).

## Data Availability

The microbial, viral abundances and environmental data that support the findings of this study are available in the *Tara* Oceans website (http://ocean-microbiome.embl.de/companion.html).

## SUPPLEMENTAL MATERIAL

Supplemental figures msystems.00539-25-s0001.docx Figures S1 to S7.

Supplemental Table S1 msystems.00539-25-s0002.xlsx Network properties of microbiomes with/without viruses across the oceans.

Supplemental Table S2 msystems.00539-25-s0003.xlsx Those nodes with top 10 degree/betweenness in the networks of microbiomes with/without viruses across the oceans.

Supplemental Table S3 msystems.00539-25-s0004.xlsx Those nodes with top 10 Zi/Pi in the networks of microbiomes with/without viruses across the oceans.

ASM does not own the copyrights to Supplemental Material that may be linked to, or accessed through, an article. The authors have granted ASM a non-exclusive, world-wide license to publish the Supplemental Material files. Please contact the corresponding author directly for reuse.
