# Supplementary material for: Correlation with viruses enhances network complexity and stability of co-occurrence prokaryotes across the oceans
Source: mSystems. 2025 Jun 13;10(7):e00539-25. doi: 10.1128/msystems.00539-25 (PMC12282058; doi:10.1128/msystems.00539-25)
Supplement: Supplemental figures — Figures S1 to S7. [file msystems.00539-25-s0001.docx]

# Correlation with viruses enhances network complexity and stability of co-occurrence prokaryotes across the oceans

Bo Wang^1^, Yantao Liang^2,3^, Kaiyue Lian^2,3^, Chuyu Zhang^2,3^, Meiaoxue Han^2,3^, Min Wang^2,3,*^, Hongbing Shao^2,3^, Andrew McMinn^4^ &. Hualong Wang^2,3, *^

^1^College of Safety and Environmental Engineering, Shandong University of Science and Technology, Qingdao, China

^2^College of Marine Life Sciences, MOE Key Laboratory of Evolution and Marine Biodiversity, Frontiers Science Center for Deep Ocean Multispheres and Earth System, and Key Lab of Polar Oceanography and Global Ocean Change, Ocean University of China, Qingdao, China

^3^UMT-OUC Joint Center for Marine Studies, Qingdao, China

^4^Institute for Marine and Antarctic Studies, University of Tasmania, Hobart, TAS, Australia

^#^ Bo Wang and Yantao Liang contributed equally to this article. Author order was determined by drawing straws.

**Correspondence:** Min Wang ([mingwang@ouc.edu.cn](mailto:mingwang@ouc.edu.cn)), Hualong Wang (wanghualong@ouc.edu.cn)

**Fig S1.** Map of the sampling stations for microbiomes and viruses across the oceans. The triangle represents those samples only collected for microbiomes.


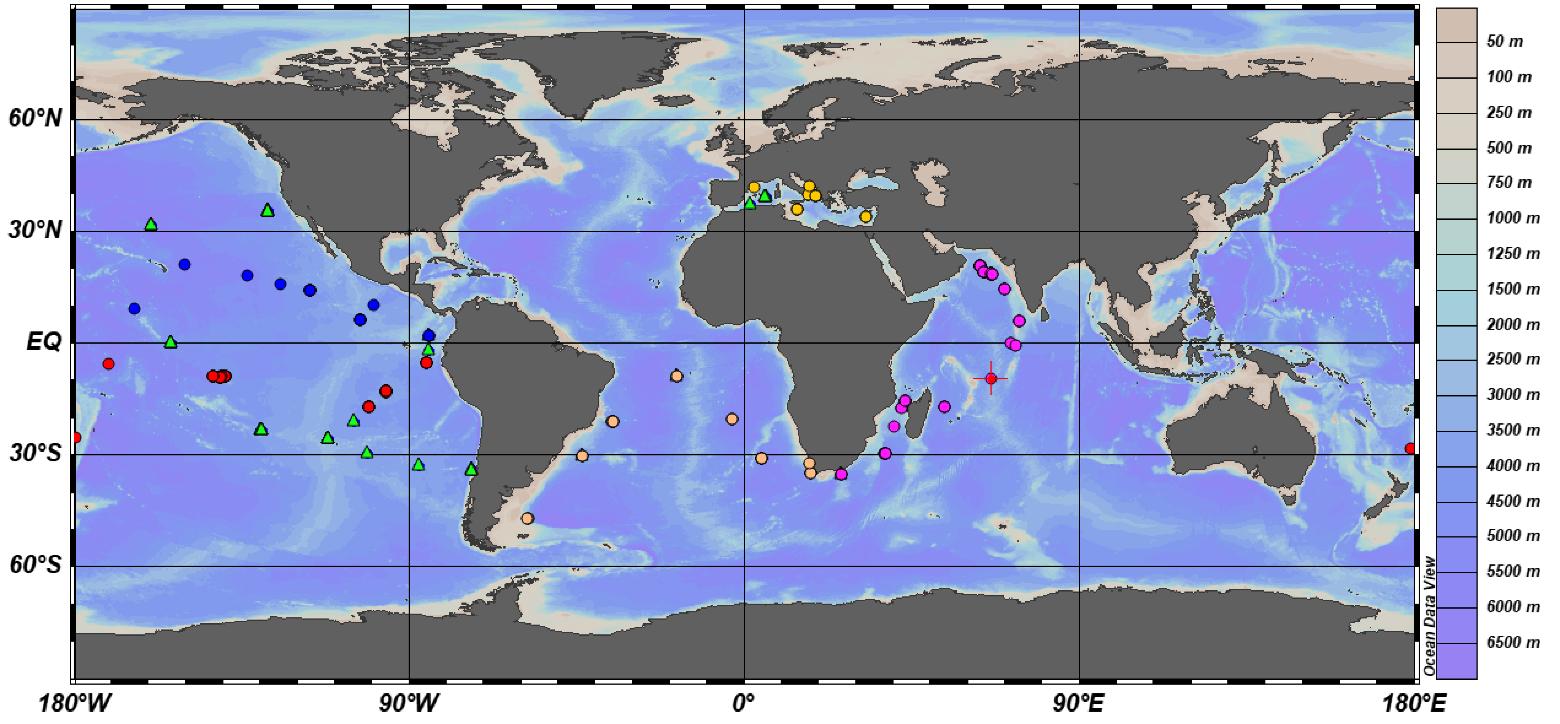

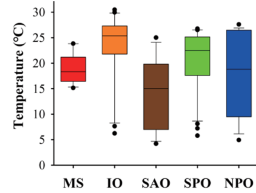

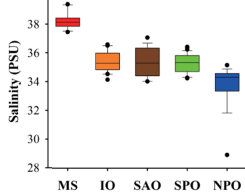


**
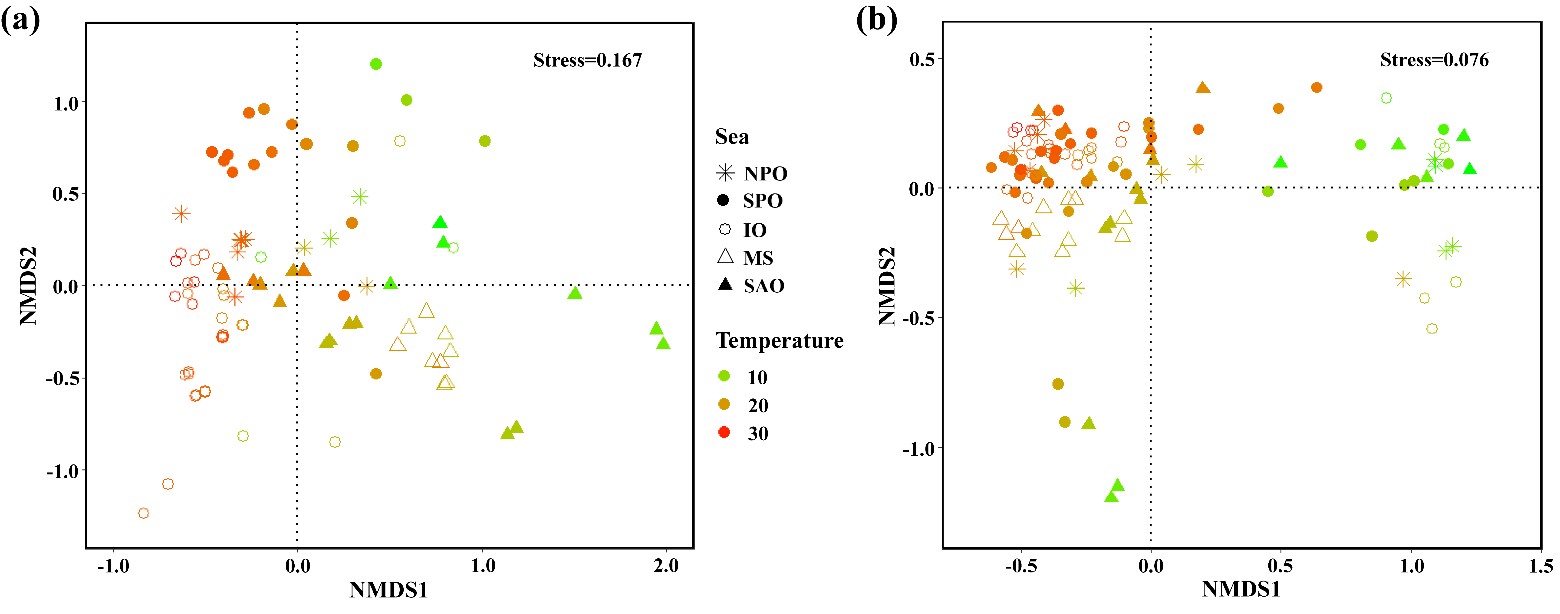
**

**Figure S2.** Nonmetric multidimensional scaling of viruses (a) and microbiomes (b) in the ocean.

**
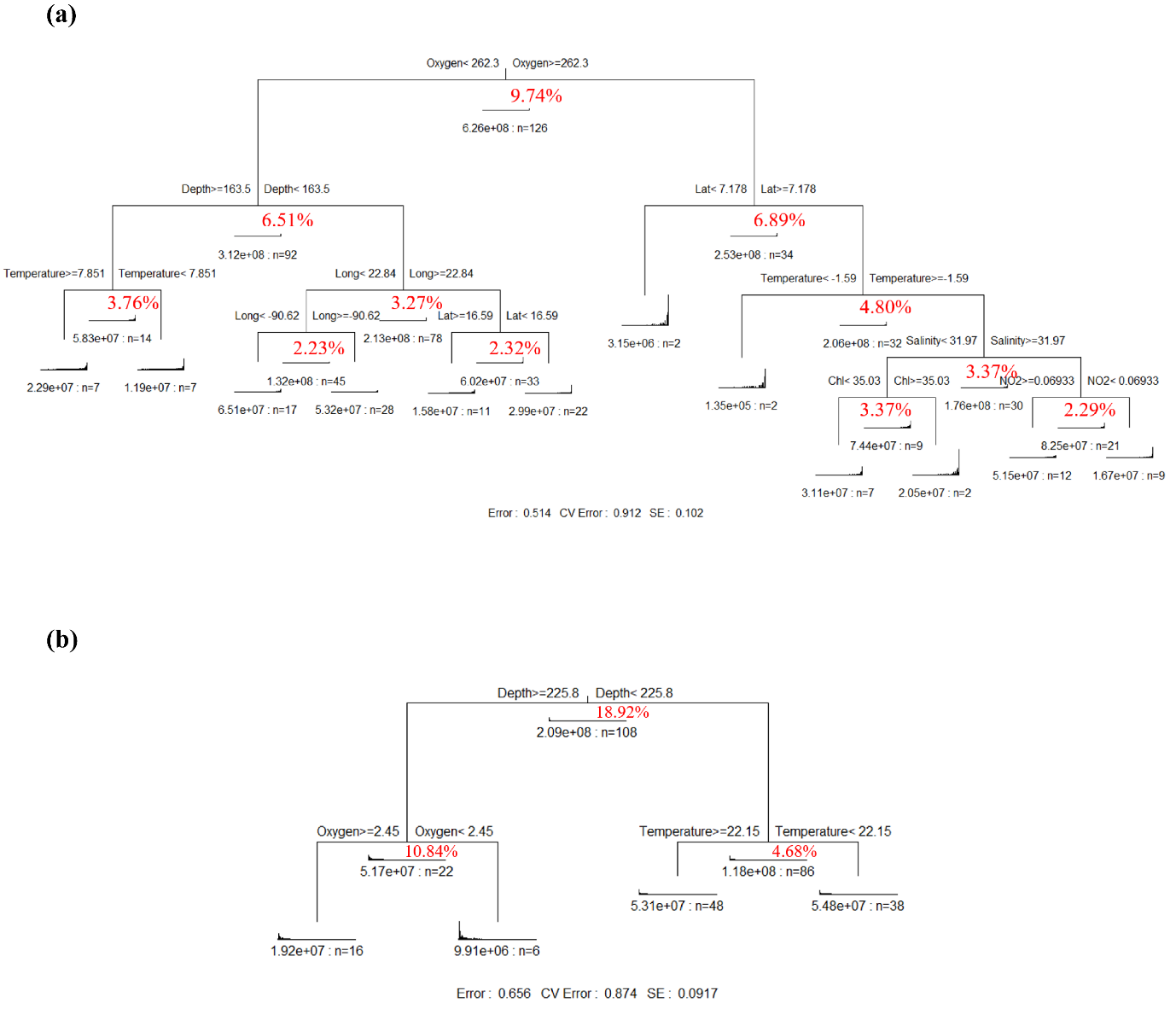
**

**Figure S3.** Multivariate regression tree analysis to show the hierarchical effects of marine environmental gradients on the viruses (a) and microbiomes (b) communities, respectively.

**
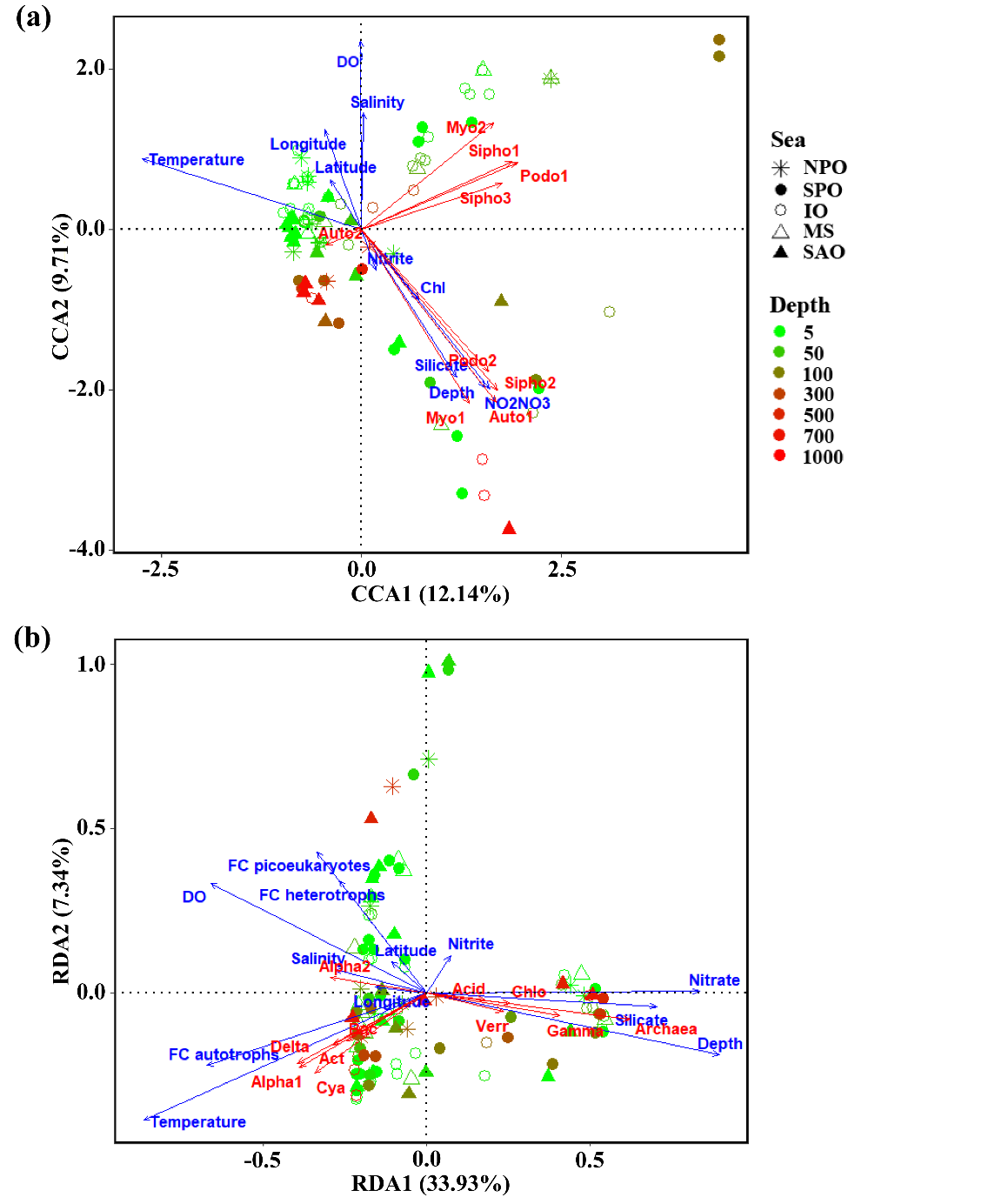
**

**Figure S4.** Relations between environmental gradients and the composition of virus (a) and microbiomes (b), respectively.


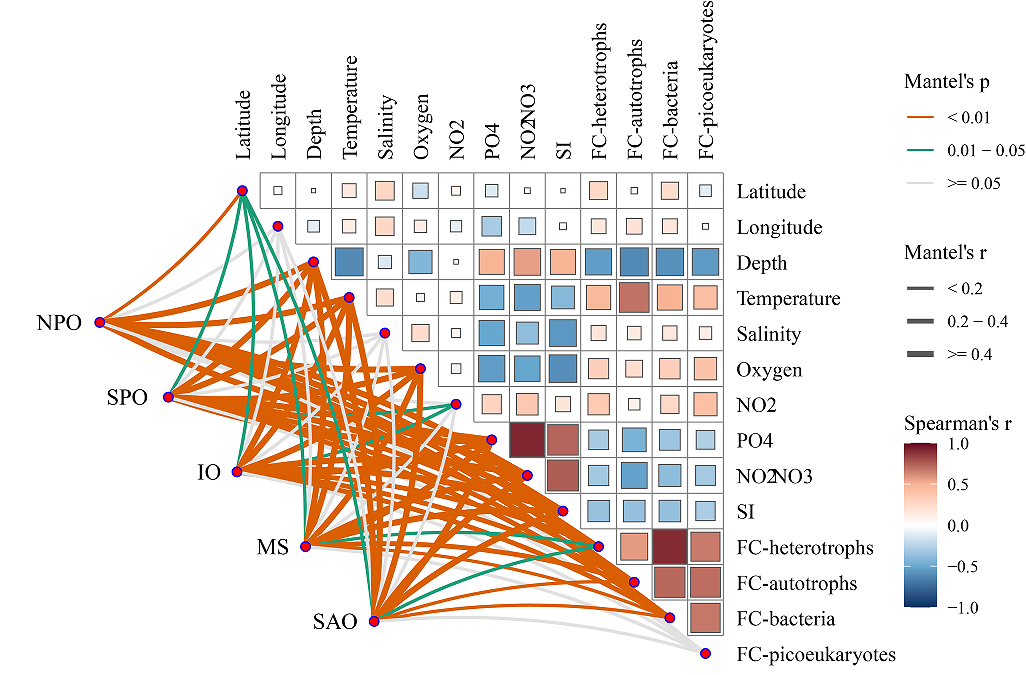
Figure S5. Mantel statistical analysis results to show the impact of environmental variation on the microbial community composition across the oceans.


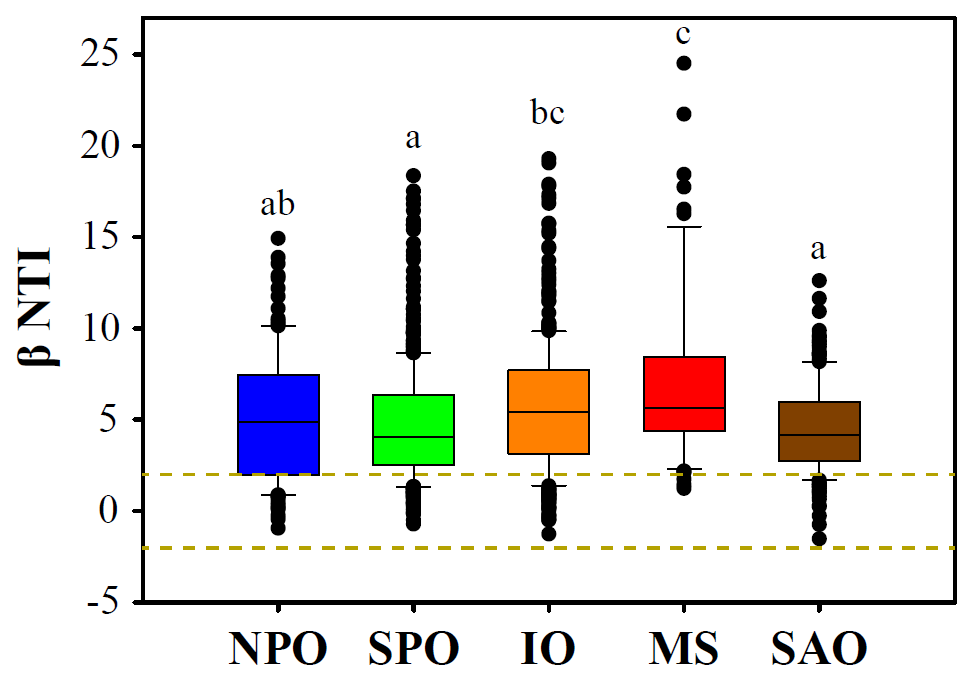


Figure S6. βNTI analysis results to show the assemble of prokaryotic microbiomes across the oceans.

**
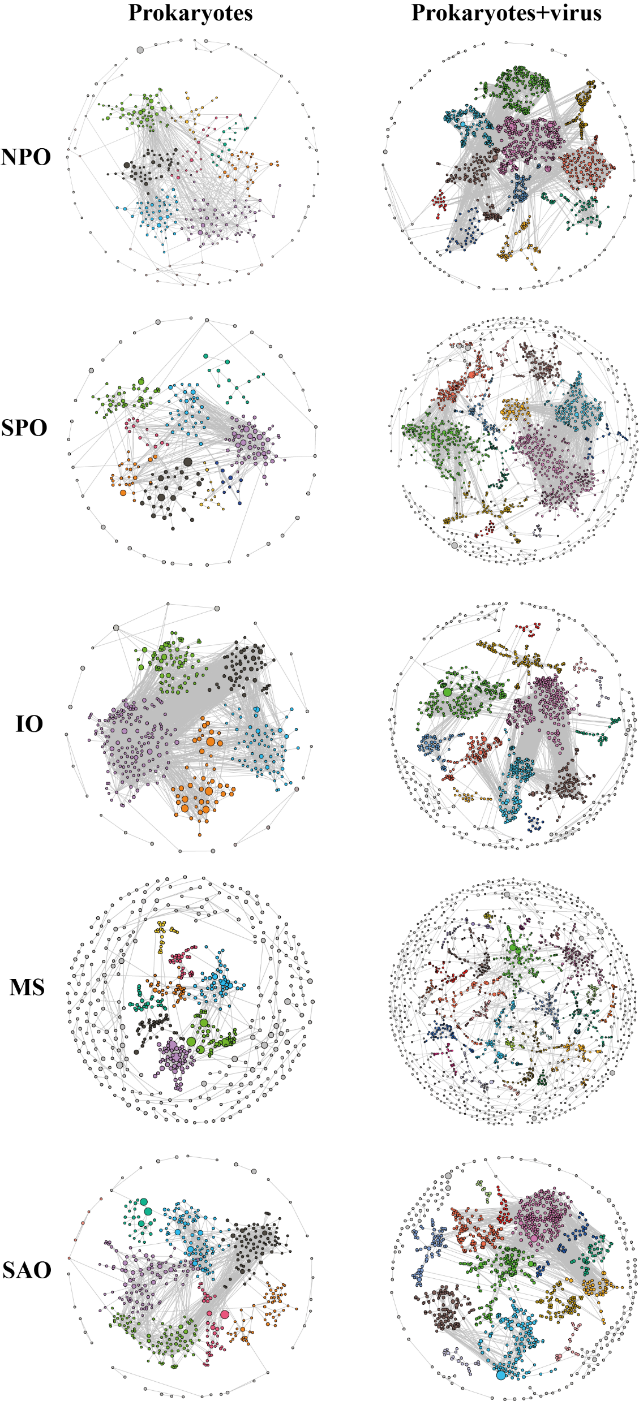
**

**Figure S7.** Co-occurrence networks of microbiomes and virus across the oceans. Color coded nodes represent organized modules. The degree of each taxa is shown by node sizes.

**Table S1.** Network properties of microbiomes with/without viruses across the oceans.

**Table S2.** Those nodes with top 10 degree/betweenness in the networks of microbiomes with/without viruses across the oceans

**Table S3.** Those nodes with top 10 Zi/Pi in the networks of microbiomes with/without viruses across the oceans.
